# Effectiveness of individual counseling for smoking cessation in smokers with chronic obstructive pulmonary disease and asymptomatic smokers

**DOI:** 10.3892/etm.2013.1463

**Published:** 2013-12-27

**Authors:** JUAN CHEN, YAN CHEN, PING CHEN, ZHIJUN LIU, HONG LUO, SHAN CAI

**Affiliations:** 1 Department of Radiology, Division of Respiratory Disease, The Second Xiangya Hospital, Central South University, Changsha, Hunan 410011, P.R. China; 2 Department of Internal Medicine, Division of Respiratory Disease, The Second Xiangya Hospital, Central South University, Changsha, Hunan 410011, P.R. China

**Keywords:** smoking cessation, individual counseling, chronic obstructive pulmonary disease

## Abstract

Few studies have examined the effect of individual counseling for smoking cessation in China. The present study evaluated the efficacy of individual counseling in patients with chronic obstructive pulmonary disease (COPD) and asymptomatic smokers. This prospective randomized study evaluated 85 smokers with COPD and 105 asymptomatic smokers with normal lung function. The individuals were randomly allocated to intervention and control groups. Subjects in the intervention group were provided with individual cognitive counseling based on face-to-face individual consultation, self-help materials and nine telephone follow-ups. Subjects in the control group were provided with simple smoking cessation advice. The smoking status for all subjects and the St. George’s Respiratory Questionnaire (SGRQ) for COPD patients were assessed at baseline, week 4 and month 6. The COPD patient exacerbations during the 6 months were recorded. In the total study population, individual counseling resulted in higher abstinence rates compared with those in the control: Intervention vs. control, 23.4 vs. 10.4% (P=0.007), respectively. Similar results were observed in the smokers with COPD: Intervention vs. control, 40.5 vs. 18.6% (P=0.027), respectively. However, for asymptomatic smokers, the effect of individual counseling was identified to be statistically insignificant: Intervention vs. control, 9.6 vs. 3.8% (P=0.230), respectively. SGRQ scores and COPD exacerbations were significantly improved in patients who abstained from smoking compared with those in the patients who failed to stop smoking. Airway obstruction, quitting motivation and individual counseling were predictors associated with smoking cessation. Airway obstruction was the most significant predictor of smoking cessation (odds ratio, 4.215; 95% confidence interval, 2.215–7.865). The results of the present study show that individual counseling is an effective method for smoking cessation, particularly in COPD patients. However, its efficacy in asymptomatic smokers requires confirmation in further studies.

## Introduction

Smoking is one of the leading causes of mortality worldwide. Over half of long-term smokers succumb to tobacco-related diseases (1). In 2010, an estimated 28.1% of adults in China (301 million; 52.9% males and 2.4% females) were current smokers and 91.8% of the smokers who attempted to quit did not use any method to assist with smoking cessation (2). Only 2.5% of males and 3.5% of females were successful at remaining abstinent for ≥24 months (3). One-third of the male population in China is likely to succumb to tobacco-related mortalities if high smoking rates persist (4). There are specific smoking cessation medications available in China; however, few smokers (5.6%) use these since there is little concern with regard to the health effects of smoking (5). Thus, the identification of an effective method to aid smoking cessation in China is urgent and cognitive intervention may be useful.

Chronic obstructive pulmonary disease (COPD) is a global health crisis with smoking as its most causative factor. Almost 50% of older smokers develop COPD (6). The prevalence of COPD in residents ≥40 years old is 8.2% in China (7). Smoking cessation is the most significant intervention in COPD management and is recommended by the Global initiative for Chronic Obstructive Lung Diseases (GOLD) (8), as it may slow down disease progression (9).

Studies from developed countries show that cognitive counseling is a cost-effective treatment for motivation and behavior-based smoking cessation (10–12). The effects shown in various groups of individuals remain ambiguous. Specific individuals consider cognitive counseling to be more effective in smokers with airway obstruction (13), while others do not consider it to be effective at all (14). Few studies concerning individual counseling for smoking cessation have been conducted in China, particularly in patients with COPD. The present prospective randomized study evaluated the efficacy of individual counseling for smoking cessation in COPD patients and asymptomatic smokers in China.

## Materials and methods

### Participants

Smoking subjects with or without COPD were recruited by residential area advertisements or from the pulmonary outpatient clinic and Physical Examination Center of the Second Xiangya Hospital (Changsha, China) over a six-month period between March 2010 and September 2010. All subjects underwent standard lung function tests with measurements of forced vital capacity (FVC) and forced expiratory volume in 1 sec (FEV_1_). COPD was defined according to the GOLD criteria (FEV_1_/FVC <70% following bronchodilatation) (8). Asymptomatic smokers were defined as smokers without respiratory symptoms (i.e., cough, sputum production or dyspnea) and with normal lung function. Enrolled subjects were >18 years-old and had smoked one or more cigarettes/day for a minimum of 100 days. Subjects with experience of smoking cessation medication and those with a history of asthma, asbestosis, silicosis, bronchiectasis or lung cancer were excluded from the study. All subjects provided informed consent approved by the medical ethics committee of the Second Xiangya Hospital.

### Study design

It is generally accepted that the motivation to quit is one of the most significant factors associated with smoking cessation (15). The smokers (COPD and asymptomatic) were divided into four categories according to their motivation to quit: No desire to quit, indifference, hoping to quit and hoping very much to quit. Smokers in each category were assigned to the intervention or control group according to the randomized digital table, where the quitting motivations were comparable between the two groups.

The intervention group was provided with individual cognitive counseling based on face-to-face individual counseling (performed at the baseline), self-help materials, as well as nine telephone calls at weeks 1, 2, 3, 4, 6 and 8 and at months 3, 4 and 5. The face-to-face individual counseling, which lasted for 20 min, was based on the five ‘A’s’ method. This relates to the harm of smoking, possible benefits of smoking cessation, methods of quitting, methods of handling withdrawal symptoms and prevention of relapses (16). For smokers with COPD, the counseling content focused on the correlation between smoking habits and COPD. The self-help materials included specific smoking cessation handbooks with smoking cessation tips. Each telephone call lasted for >10 min and was designed to further promote smoking cessation and help smokers conquer issues that occurred during smoking cessation. The control group was provided with smoking cessation advice. All interventions were conducted by two doctors with experience of professional smoking cessation treatment.

The main assessments were performed at the baseline, week 4 and month 6. Nicotine dependence was assessed using the Fagerström Test for Nicotine Dependence (FTND). The St. George Respiratory Questionnaire (SGRQ) for measuring health-related quality of life and exacerbation times during the 6 months was recorded for COPD patients. An exacerbation was defined according to the GOLD parameter, specifically associated with acute events characterized by a worsening of the patient’s respiratory symptoms beyond normal day-to-day variations, leading to a change in medication (8). The abstinence was defined as a self-reported sustained abstinence from week 4 to month 6. The self-reported abstinence at week 4 and month 6 was verified by an exhaled carbon monoxide level of <10 ppm.

### Statistical analysis

The minimum total sample was 152 participants. The expected to the success rates were 25% for the intervention group, compared with 5% for the control group, with a two tailed significance level of 0.05 and a power of 80%. Statistical analyses were performed using SPSS 17.0 statistical software (SPSS, Inc. Chicago, IL, USA). P<0.05 was considered to indicate a statistically significant difference. The normally distributed quantitative data was analyzed using t-tests, non-normally distributed data with Wilcoxon Mann-Whitney U-tests and categorical variable data with χ^2^ tests. Predictors of outcome were analyzed with multinomial logistic regression.

## Results

### Baseline characteristics

A total of 85 COPD patients and 105 asymptomatic smokers were included in the study. Twelve smokers (5 COPD and 7 asymptomatic) withdrew during the 6-month period. The most common reason for withdrawal was attributed to poor compliance. The withdrawers were regarded as smokers and were included in the outcome analyses. Table I lists the baseline characteristics of all subjects. The baseline characteristics were kept well-balanced between the intervention and the control groups; they were also balanced between the intervention and the control group for COPD and asymptomatic smokers. There were certain significant differences in baseline characteristics between the COPD patients and asymptomatic smokers. Age, pack-years and quitting motivations were significantly higher in COPD patients than in asymptomatic smokers and the educational level in the COPD patients was lower than that in asymptomatic smokers. The severity of COPD was classified according to the predicted FEV_1_ percentage. The following percentages were observed in terms of COPD grading: 11% subjects were mild (FEV_1_ ≥80% predicted), 46% were moderate (FEV_1_ ≥50–80% predicted), 33% were severe (FEV_1_ ≥30–50% predicted) and 10% were very severe (FEV_1_ <30% predicted). A full representation is shown in Table II.

### Smoking cessation outcomes

**Figure 1:**
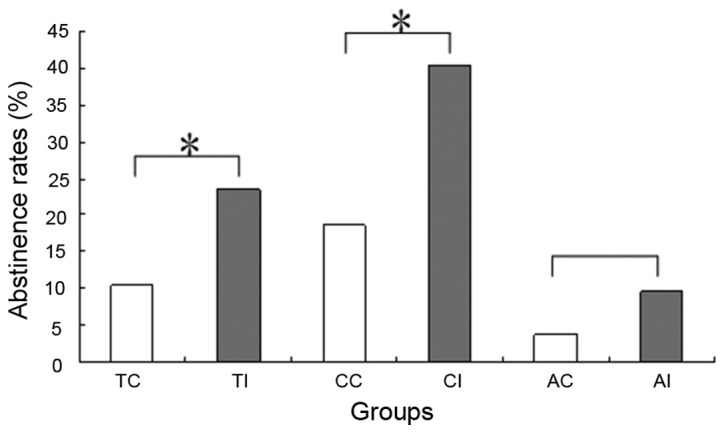
Abstinence rates in various groups. ^*^P<0.05, as indicated. TC, total smokers control; TI, total smokers intervention; CC, COPD control; CI, COPD intervention; AC, asymptomatic control; AI, asymptomatic intervention; COPD, chronic obstructive pulmonary disease.

Fig. 1 lists smoking cessation outcomes. When smokers with and without COPD were considered together, the abstinence rates were higher in the intervention group than in the control group (23.4 vs. 10.4%; P=0.007). The result is similar to that based exclusively on smokers with COPD (40.5 vs. 18.6%; P=0.027). However, the effect of individual counseling in asymptomatic smokers was statistically insignificant (9.6 vs. 3.8%; P=0.230). Table II lists the various abstinence rates in smokers who have different spirometric results. Smokers with COPD were more likely to quit smoking (29.4%) than those with normal spirometry (6.7%; P<0.001). Although higher COPD grades correlated with a higher abstinence rate, the differences in abstinence rates among the mild, moderate, severe and very severe COPD groups were statistically insignificant (22.2, 25.6, 31.0, 50.0%, respectively; P=0.540).

### Health outcomes

This analysis was based on the COPD participants. Only the proportion of patients who had an improvement of four or more SGRQ score units was used in the calculation, as this group showed significant clinical changes in the quality of life. Of the COPD patients who abstained from smoking, 36.0% had significant improvements in the SGRQ total scores; this was significantly higher than the 13.3% in patients who failed to stop smoking (P=0.037). During the six-month period, there was an average of 0.61 exacerbations in the COPD patients who abstained from smoking, compared with 1.21 exacerbations in the patients who continued to smoke (Z, −3.32; P<0.001).

### Predictors of smoking cessation outcomes

Multinomial logistic regression was used to analyze whether the abstinence outcome was associated with various predictors (Table III). Individual counseling, COPD and quitting motivation were found to be independent predictors of abstinence, where COPD was the most significant (odds ratio, 4.215; 95% confidence interval, 2.215–7.865). Other factors, including age, gender, daily cigarettes, pack-years, FTND, educational background and SGRQ, were included in the initial regression model. However, they were not found to have a statistically significant association with abstinence.

## Discussion

The present study is, to the best of our knowledge, the first randomized controlled trial in China that has demonstrated the efficacy of individual counseling for smoking cessation in smokers with COPD and asymptomatic smokers. The most significant observation was that individual counseling was an effective method for smoking cessation. This was particularly true in smokers with COPD.

The results of the present study correlated with previous studies evaluating the efficiency of individual counseling for smoking cessation in developed countries. Bednarek *et al* (13), in Poland, recruited 4,494 smokers; all these smokers received simple smoking cessation advice while 1,177 subjects with airway obstruction were informed that they had COPD and that smoking cessation would halt the lung disease progression. One year later, the sustained smoking cessation rate in those with airway obstruction was 16.3%, compared with 12.0% in those with normal spirometric parameters (P=0.0003) (12). Stratelis *et al* (17), in Sweden, obtained a similar result. In this study, 512 smokers enrolled and received annual spirometry and brief smoking cessation advice, followed up by a personal letter from a physician. Three years later, 25% of smokers with COPD were smoke-free for ≥1 year, compared with 7% of smokers with normal lung function (P<0.001). These studies and the results of the present study indicate that individual counseling is efficacious in helping smokers to quit and its efficiency in COPD patients was more marked. However, a study by Willemse *et al* (14) in the Netherlands used group meetings for smoking cessation and reported a 42% (16/38) abstinence rate in COPD patient smokers, in comparison to a 68% (17/25) abstinence rate in healthy smokers. The authors did not offer the statistical analysis for these results. One explanation may be that the healthy smokers that were recruited had a higher motivation for smoking cessation. The authors did not assess the motivation of their subjects. Toljamo *et al* (15), in Finland, enrolled 584 smokers; the study provided individual counseling to all individuals. The study reported a 5.4% sustained abstinence rate in smokers without COPD, compared with 10.6% in patients with COPD (P=0.125); success in quitting was not identified to be associated with airway obstruction. However, there was a clear difference between their study and the present study. Spirometry was conducted at the end of their study. Therefore, the COPD patients were not informed of their illness. In comparison, the patients in the present study, who received counseling, found that an awareness of the correlation between smoking habits and COPD may be useful for smoking cessation.

Improved cessation rates were found in smokers who had COPD. The factors leading to this result included several components. First, the quitting motivations were higher in COPD patients than in general smokers. The majority of patients with COPD suffered from a cough and dyspnea, which are widely considered to be harmful effects of smoking. Concern for personal health was the most popular reason for considering smoking cessation in China (5). Secondly, positive spirometric results and a COPD diagnosis show patients the harmful effects of smoking and the necessity of smoking cessation. The United States National Lung Health Education Program previously recommended spirometric testing for increasing the motivation of smokers to quit (18). Parkes *et al* (19) used ‘lung age’, according to spirometric results, in order to encourage smoking cessation; their study reported that independently verified rates of quitting at 12 months in the intervention and control groups were 13.6 and 6.4% (P=0.005), respectively. In addition, smoking is a social behavior in China. Individuals often share cigarettes with each other, making it difficult for asymptomatic smokers to stop smoking. Smokers with COPD may reject cigarettes offered to them due to their disease.

The goals of clinical control for COPD patients included improvement of exercise tolerance, emotional function, prevention of disease progression and minimization of symptoms, all of which may lead to an increased quality of life (8). Despite continuous airway inflammation in ex-smokers with COPD (20), smoking cessation is an effective method of reducing the progress of the disease by slowing down the annual FEV_1_ decline rate (9) and it may result in an increase of quality-adjusted life-years for COPD patients (10). The dyspnea and cough symptoms that COPD patients often experience may be improved following smoking cessation (21). The health outcomes in the present study demonstrate statistically significant improvements in SGRQ total scores and COPD exacerbations in patients with COPD who abstained from smoking, demonstrating that smoking cessation may result in increased health benefits for COPD patients.

A number of factors are involved in smoking cessation. Studies from Western countries have shown that health status and economic reasons are the most commonly associated factors (22,23). In addition, smoking abstinence is associated with old age, male gender, high income, high levels of education, low nicotine dependence and smoke-free family policies (15,24). A study from South Africa showed that clinical interventions and smoke-free family policies may increase the rate of smoking cessation (25). Previous studies in China observed that the interest in quitting among smokers is affected by past quitting experiences, nicotine dependence, health concerns and attitudes towards smoking (26,27). The observations of the present study confirmed previous observations showing that motivation and health status were the most significant factors affecting the result of smoking cessation. It was found that individual counseling was another significant factor. Demographic characteristics and nicotine dependence were not identified to be independent factors for smoking cessation, possibly owing to the small sample size.

There are several limitations in the present study. The majority of COPD patients were recruited from the outpatient clinic and therefore possibly had more severe clinical symptoms than the typical patient with COPD. Thus, the patients may be more motivated to quit smoking. In addition, the majority of the subjects in this study were from Hunan Province in China. Hence, the study population may not be a true representation of all smokers in China.

In conclusion, individual smoking cessation counseling is an effective method for helping COPD patients to quit and may result in increased health benefits. Health professionals should provide cognitive counseling to smokers to help them quit smoking. Quitting motivation, healthy status factors and individual counseling are significant factors associated with smoking cessation results. The individual counseling efficiency for asymptomatic smokers remains uncertain and further nationwide multicenter studies are required to investigate this further in the future.

## Figures and Tables

**Table I:** Participant characteristics.

			COPD		Asymptomatic smokers	
				
Characteristics	Intervention group, n=94	Control group, n=96	Intervention group, n=42	Control group, n=43	Intervention group, n=52	Control group, n=53
Age, years	50.8±13.9	50.3±13.2	61.4±8.6	61.6±7.7	41.7±10.1^a^	41.6±9.8^b^
Male:female	91:3	93:3	41:1	41:2	50:2	52:1
Pack-years	31.6±18.8	32.6±20.4	40.2±18.2	44.3±15.9	23.7±15.4^a^	24.0±19.4^b^
Daily cigarettes	19.0±10.3	20.6±12.7	17.5±8.4	17.2±9.6	22.4±10.6	23.6±13.9
Carbon monoxide, ppm	15.1±8.2	16.1±8.2	14.2±7.3	14.1±8.5	17.5±8.4	18.3±9.2
FTND, points	4.1±2.4	4.2±2.3	4.0±2.0	4.1±2.5	4.2±2.1	4.3±2.4
Education, n						
Junior high school or less	40	34	28	20	12	14
Senior high school	23	23	10	14	13	9
College or more	31	39	4	9	27	30
Quitting motivation, n						
No desire to quit	17	18	3	3	14	15
Indifference	27	26	5	5	22	21
Hoping to quit	37	39	23	24	14	15
Hoping very much to quit	13	13	11	11	2	2

a P<0.05, vs. COPD intervention group;

b P<0.05, vs. COPD control group.

Pack-years were calculated by multiplying the number of packs of cigarettes smoked per day by the number of years the individual had smoked. COPD, chronic obstructive pulmonary disease; FTND, Fagerström Test for Nicotine Dependence.

**Table II:** Abstinence rates stratified by baseline spirometric results.

		COPD				
		
Variable	Normal	All	Mild	Moderate	Severe	Very severe
Subjects	105	85	9	39	29	8
Abstainers	7	25	2	10	9	4
Abstinence rates, %	6.7	29.4	22.2	25.6	31.0	50.0
P-value	<0.001^a^	0.540^b^				

a P-value compares abstinence rates between asymptomatic smokers and COPD patients;

b P-value compares abstinence rates among various grades of COPD patients.

COPD, chronic obstructive pulmonary disease.

**Table III:** Predictors of smoking cessation in the logistic regression model.

Predictors	B	P-value	OR	95% CI
Airway obstruction	1.652	0.000	4.217	2.215–7.865
Individual counseling	1.133	0.007	3.104	1.369–7.042
Quitting motivation	1.146	0.000	3.145	1.780–5.557

OR, odds ratio; CI, confidence interval.
